# Working from home, quality of life, and perceived productivity during the first 50-day COVID-19 mitigation measures in Austria: a cross-sectional study

**DOI:** 10.1007/s00420-021-01692-0

**Published:** 2021-04-20

**Authors:** Jakob Weitzer, Kyriaki Papantoniou, Stefan Seidel, Gerhard Klösch, Guido Caniglia, Manfred Laubichler, Martin Bertau, Brenda M. Birmann, Carlo C. Jäger, Lukas Zenk, Gerald Steiner, Eva Schernhammer

**Affiliations:** 1 Department of Epidemiology, Center for Public Health, Medical University of Vienna, Vienna, Austria; 2 Department of Neurology, Medical University of Vienna, Vienna, Austria; 3 Institute for Sleep-Wake-Research, Vienna, Austria; 4 Konrad Lorenz Institute for Evolution and Cognition Research (KLI), Klosterneuburg, Austria; 5 School of Life Sciences, Arizona State University, Tempe, AZ USA; 6 Santa Fe Institute, Santa Fe, NM USA; 7 Complexity Science Hub, Vienna, Austria; 8 Institut Für Technische Chemie, TU Bergakademie Freiberg, Freiberg, Germany; 9 Channing Division of Network Medicine, Department of Medicine, Brigham and Women’s Hospital and Harvard Medical School, Boston, MA USA; 10 Academy of Disaster Reduction and Emergency Management, School of Geography, Beijing Normal University, Beijing, 100875 China; 11 Global Climate Forum, 10178 Berlin, Germany; 12 Department for Knowledge and Communication Management, Faculty of Business and Globalization, Danube University Krems, Krems, Austria; 13 Department of Epidemiology, Harvard T.H. Chan School of Public Health, Boston, MA USA

**Keywords:** Telework, Work from home, Occupational health, Quality of life

## Abstract

**Objectives:**

To explore changes in quality of life and perceived productivity, focusing on the effects of working from home during the first COVID-19 50-day mitigation period in Austria.

**Methods:**

We conducted an Austrian-representative online survey (*N* = 1010) of self-reported life- and work-related changes during the first COVID-19 50-day mitigation period (March 16 through May 1 2020) compared to the situation before. We used multinominal logistic regression models to identify correlates of improved/decreased quality of life in the entire sample, and of improved/decreased productivity in a subsample of the working population (*N* = 686). We also calculated age- and multivariable-adjusted ORs and 95% CIs of an improved/decreased quality of life and an improved/decreased productivity by work from home status.

**Results:**

During the COVID-19 mitigation period, quality of life improved in 17.5%, but decreased in 20.7% of the general Austrian population; perceived productivity at work increased in 12.7%, but decreased in 30.2% of the working population. Working from home during the mitigation period was associated with an increased quality of life (vs. none, partially: OR 2.07, 95% CI 1.09–3.91; all the time: 3.69, 1.86–7.29). In contrast, perceived productivity seemed to decrease when people worked from home (vs. none, partially: 1.42, 0.86–2.35; all the time: 1.48, 0.85–2.58). Working from home and related benefits were not equally distributed among gender, age, and educational attainment.

**Conclusions:**

A transition to more flexibility of workplace and working hours for employees could have important positive consequences for family and professional life, for stakeholders, for public health, and ultimately for the environment.

**Supplementary Information:**

The online version contains supplementary material available at 10.1007/s00420-021-01692-0.

## Introduction

Across the globe, confinement measures to curb the spread of COVID-19 contributed to unhealthy lifestyle behaviors (Ammar et al. 2020a, b; Deschasaux-Tanguy et al. 2020; Reyes-Olavarría et al. 2020) and negative health outcomes, including important increases in the prevalence of psychosocial and emotional disorders (Ammar.et al. 2020b). However, some positive consequences in the context of “One Health”, which includes all aspects of human, environmental, and animal health (Laubichler 2020), have also been documented, e.g., for the environment (Zambrano-Monserrate et al. 2020) and for certain health outcomes in some sections of society. Importantly, specific COVID-19 mitigation strategies differed across countries and differentially affected specific sections of societies. Thus, the overall implications of COVID-19 mitigation measures for public and environmental health remain uncertain.

A 50-day mitigation period in response to the first COVID-19 wave in Austria started on March 16, 2020 (Republik Österreich 2020a) with restrictions lifted on May 1, 2020 (Republik Österreich 2020b). The policy included a ban on entering public places with only five exceptions: errands to cover necessary basic needs, professional activity (i.e., by essential workers such as health care workers), care and assistance for those in need of support, exercise outdoors alone or with people living in the same household, and averting danger to life, limb or property (Republik Österreich 2020a). Schools and kindergardens were closed (Kurier 2020), and day care was provided only for essential workers, e.g., health care workers, who could not allocate other resources for childcare.

In Austria, the prevalence of mental health problems increased during the mitigation period, with younger age, female gender, unemployment and low income as the driving risk factors (Pieh et al. 2020). In addition, a decrease of in-person psychotherapy was not compensated by increases in remote psychotherapy (Probst et al. 2020). In other countries, a few positive outcomes of COVID-19 mitigation measures have been reported, for example, eating more healthily than before (Deschasaux-Tanguy et al. 2020; Reyes-Olavarría et al. 2020). Before the emergence of the COVID-19 pandemic, studies mainly reported negative effects of quarantine measures on mental health, suggesting potentially long-lasting consequences (Brooks et al. 2020). However, COVID-19 measures came with a novelty for a large part of the population: work from home.

Evidence before pandemic times suggests that work from home can improve life and work in several ways. People who chose to work from home reported to enjoy greater flexibility in relation to work, leisure activities, and family (Laegran 2008). More control and choice in work is expected to improve wellbeing (Joyce et al. 2010), and working from home has been linked to higher job satisfaction (Troup and Rose 2012). Finally, Eurofound concluded that telework has mainly positive effects on individual performance (Eurofound 2017), although working from home entails problems too, such as unclear boundaries between work and private life (Palumbo 2020; Palumbo et al. 2020; Vittersø et al. 2003) and lack of interpersonal relationship of colleagues (Webster and Wong 2008).

In early times of the pandemic, the transition to working from home was associated with a decrease in physical and mental health in a North American sample although reported associations might be the consequence of overall mitigation measures (Xiao et al. 2020). In a Japanese sample, working from home was associated with less depressive symptoms among men who worked long hours and among women in general (Sato et al. 2020), while a study among software engineers indicated a neutral effect of working from home on their well-being and productivity (Russo et al. 2020). In Austria, approximately 25% of the working population worked from home during the first mitigation period, and 72% of those stated that they preferred working from home once the pandemic ends (TQS Research & Consulting 2020). Furthermore, a survey among 526 Austrian companies revealed that 54% plan to maintain post-pandemic work-from-home options for some employees (Land Niederösterreich 2020). In sum, it can be expected that working from home comes with positive and negative side effects even during pandemic times, and the new high in people working from home warrants to further explore how working from home might affect work and life.

Therefore, the objective of this study was to explore changes in quality of life and perceived productivity, with a specific focus on the effects of working from home during the first COVID-19 50-day mitigation period in Austria.

### Methods

### Study design and setting

Between June 3 and June 23, 2020, we conducted an online survey assessing changes in lifestyle and work-related characteristics with specific reference to the time period during the COVID-19 mitigation measures in Austria (March 16 to May 1, 2020) among 1010 Austrians randomly selected from an Online-Panel quota sampled to represent the age (18–65 years), sex and county distribution of Austria’s general population. To assess changes in lifestyle, quality of life and perceived productivity, participants rated on a 5-point Likert scale ranging from “decreased importantly” to “increased importantly” whether aforementioned variables had changed during the mitigation period compared to before the pandemic. The survey, which included 81 questions and took approximately 30 minutes to complete, was implemented by Interrogare (www.interrogare.de), a health care research institute based in Germany. Participation was voluntary and anonymous, and informed consent was implied through participation.

### Variables

In addition to demographic and lifestyle variables, participants indicated on a Likert scale [decreased importantly, decreased, no change, improved, improved importantly] if their quality of life and, among those in the work force, perceived productivity at work, had changed during the COVID-19 mitigation period compared to the time before the mitigation period. Participants reported if they were working from home during the mitigation period [not working from home, working partially from home, working from home all the time]. The covariates, which included age [< 30, 30–39, 40–49, 50–59, ≥ 60 years], gender [female, male], educational attainment [high school or less, university entry exam, university degree], citizenship [Austrian, other], race [Caucasian/White, other], region of residence [West, South, East], residential area [urban, rural with < 50,000 inhabitants, rural with at least 50,000 inhabitants], marital status [single/married/in partnership, divorced/widowed], size of household including oneself [single, 2, 3, 4 + persons], number of children [none, 1/2/3 +], having to take care of children younger than 6 years at home [yes, no, none that age], having to take care of children ages 6–16 at home [yes, no, none that age], current work status [employed (including self-employed) full-time, employed (including self-employed) part-time, retired, unemployed, student or in training], financial strain during mitigation period [none, some, high, very high], dispositional optimism assessed using the validated Life Orientation Test‐Revised (LOT‐R) (Scheier et al. 1994) [low, high], job loss [yes, no] and short-time work [no, yes, not employed before the mitigation period], were self-reported. Short-time work, a policy to help retain jobs, was implemented in Austria at the beginning of the COVID-19 pandemic and entailed having companies reduce employee work hours while continuing to pay almost full salary, with the government subsidizing a major portion of the salaries.

### Statistical methods

We used multinominal logistic regression models to calculate age- and multivariable-adjusted odds ratios (ORs) and 95% confidence intervals (95% CI) of improved/decreased quality of life in the entire sample, and of improved/decreased productivity in a subsample comprising only those who had been employed before the mitigation period (working population: *N* = 686). In the latter subset, we also calculated age- and multivariable-adjusted ORs and 95% CIs of an improved/decreased quality of life and an improved/decreased productivity by work from home status (not, part of the time, all the time). Our multivariable models considered the covariables listed above as confounders. A two-sided significance level (*α* = 0.05) and STATA (version 14.1, 2015, StataCorp LP) were used for all analyses.

### Results

### Sample characteristics

Of 1010 respondents to the survey, three with missing information on gender were excluded, leaving 1007 participants for our analyses. Fifty-five percent of survey participants were women, and approximately 91% were Austrian citizens. Of all participants, 65.3% (men, 68.8%; women, 62.0%) were part- or full-time employed (including those self-employed) and 4.6% had lost their job during the mitigation period (men, 4.8%; women, 4.3%). Men were older and more frequently reported having received only basic education. 31.8% of the whole sample had not been employed (or self-employed) before the mitigation period. During the mitigation period, 17.7% of the sample were not working from home, 29.5% were working from home part of the time and 21.0% all the time (Table 1). In the working sample, those working from home were more frequently men (75.1% vs. 72.9% working from home), younger (< 30 years, 82.6% working from home; 30–49 years, 78.1%; ≥ 50 years, 61.1%), and participants who had received higher education (high school or less, 57.7% working from home; University entering exam, 80.8%; University degree, 85%).

**Table 1:** Characteristics of the study sample (*N* = 1007)

	Men (*N* = 499)	Women (*N* = 508)	Total (*N* = 1007)
	*N* (%)	*N* (%)	*N* (%)
Age, years			
< 30	98 (19.6)	133 (26.2)	231 (22.9)
30–39	84 (16.8)	108 (21.3)	192 (19.1)
40–49	133 (26.7)	116 (22.8)	249 (24.7)
50–59	120 (24.1)	114 (22.4)	234 (23.3)
≥ 60	64 (12.8)	37 (7.3)	101 (10.0)
Highest education			
High school or less	198 (39.7)	165 (32.5)	363 (36.1)
Matura (University entry exam)	166 (33.3)	200 (39.4)	366 (36.3)
University degree	135 (27.0)	143 (28.1)	278 (27.6)
Citizenship			
Austrian	459 (92.0)	455 (89.6)	914 (90.8)
Other EU country	24 (4.8)	39 (7.7)	63 (6.2)
Non-EU country	16 (3.2)	14 (2.7)	30 (3.0)
Region of residence			
Burgenland	18 (3.6)	19 (3.7)	37 (3.7)
Carinthia	30 (6.0)	32 (6.3)	62 (6.2)
Lower Austria	96 (19.3)	100 (19.7)	196 (19.5)
Salzburg	25 (5.0)	23 (4.5)	48 (4.8)
Styria	76 (15.2)	58 (11.4)	134 (13.3)
Tyrol	36 (7.2)	45 (8.9)	81 (8.0)
Upper Austria	73 (14.6)	74 (14.6)	147 (14.6)
Vienna	124 (24.9)	139 (27.4)	263 (26.1)
Vorarlberg	21 (4.2)	18 (3.5)	39 (3.8)
Current work status			
Employed full time^a^	305 (61.1)	229 (45.1)	534 (53.0)
Employed part time^a^	38 (7.7)	86 (16.9)	124 (12.3)
Retired	55 (11.0)	48 (9.4)	103 (10.2)
Unemployed	46 (9.2)	44 (8.7)	90 (9.0)
Student, in training, civil service	55 (11.0)	101 (19.9)	156 (15.5)
Job loss during lockdown	24 (4.8)	22 (4.3)	46 (4.6)
Working from home during lockdown			
No	87 (17.4)	91 (17.9)	178 (17.7)
Partially	155 (31.1)	142 (28.0)	297 (29.5)
All the time	108 (21.6)	103 (20.2)	211 (21.0)
Not employed before lockdown	149 (29.9)	172 (33.9)	321 (31.8)

^a^Includes self-employed participants

### Prevalence of changes in quality of life and perceived productivity

**Fig. 1:**
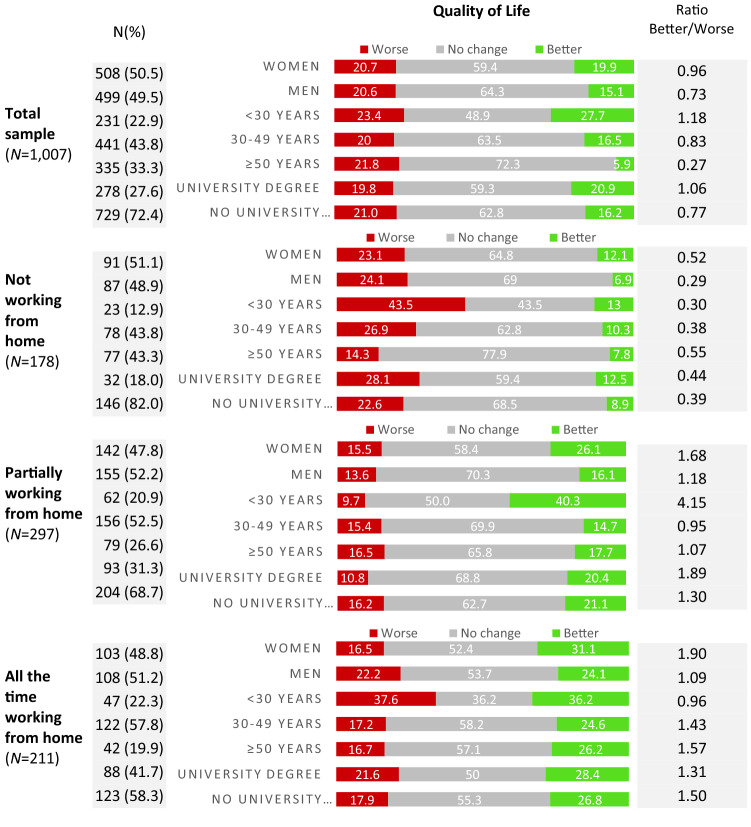
Changes in quality of life during the COVID-19 mitigation period, in the entire Austrian population sample and by work from home status in the working population sub-sample

During the COVID-19 mitigation period in Austria, quality of life improved for 17.5% (men, 15.1%; women, 19.9%) and decreased for 20.7% (men, 20.6%; women, 20.7%) of the overall Austrian population sample (Fig. 1). Younger and highly educated participants reported an improved quality of life more often than older or less educated participants. In addition, participants reported improved quality of life more frequently if they worked from home than if they did not work from home—a trend which was seen irrespective of age group and level of educational attainment (Fig. 1).

Productivity at work improved in 12.7% (men, 13.4%; women, 11.9%) and decreased in 30.2% (men, 27.4%; women, 33.0%) of the working population sample. Younger individuals reported decreased work productivity more frequently than older persons, and participants with a higher educational status reported improved productivity more frequently than those with less education (Supplementary Fig. 1). Men, but not women, who worked from home during the mitigation period reported an increased productivity more frequently than those who did not work from home (none, 8.1%; part of the time, 11.6%; all the time, 20.4%). Increased productivity was also reported more frequently by highly educated participants who worked from home than by those who did not (none, 3.1%; part of the time, 18.3%; all the time, 21.6%; Supplementary Fig. 1). Participants reporting an improved quality of life more frequently reported an improved perceived productivity (19.7% vs. 12.7%) compared to those reporting a decreased quality of life.

Older participants, men, and persons not working from home were most likely to report no changes in quality of life or perceived productivity (Fig. 1 and supplementary Fig. 1).

### Correlates of positive and negative changes

In multivariable-adjusted models, among men, younger age, living in an urban area, being in short-time work, and experiencing high financial strain significantly and positively correlated with improved quality of life. Furthermore, men with an university degree, compared to those less educated (high school or less), (OR 1.58, 95% CI 0.78–3.22) and men married or in a partnership, compared to single men (OR 1.78, 95% CI 0.80–3.94), appeared more likely to report an improved quality of life. Men taking care of children between 6 and 16 years were more likely to report a decreased quality of life compared to men without children this age. Furthermore, being in short-time work and experiencing a higher financial strain significantly and positively correlated with decreased quality of life in men (Table 2).

**Table 2:** Predictors of decreased/improved quality of life in the entire Austrian general population sample (*N* = 1007)

	Change in quality of life									
	Men (*N* = 499)					Women (*N* = 508)				
	No change (*N* = 321)	Decreased (*N* = 103)	OR (95% CI)^a^	Improved (*N* = 75)	OR (95% CI)^a^	No change (*N* = 302)	Decreased (*N* = 105)	OR (95% CI)a	Improved (*N* = 101)	OR (95% CI)^a^
	*N* (%)	*N* *(%)*		*N* (%)		*N* (%)	*N* *(%)*		*N* (%)	
Age, years										
< 30	50 (15.6)	26 (25.2)	1	22 (29.3)	1	63 (20.9)	28 (26.7)	1	42 (41.6)	1
30–39	51 (15.9)	15 (14.6)	0.53 (0.21–1.34)	18 (24.0)	0.66 (0.28–1.52)	61 (20.2)	19 (18.1)	0.50 (0.22–1.16)	28 (27.7)	0.70 (0.34–1.43)
40–49	93 (29.0)	29 (28.2)	0.82 (0.34–1.98)	11 (14.7)	**0.22 (0.08–0.58)**	75 (24.8)	25 (23.8)	0.57 (0.25–1.30)	16 (15.8)	**0.35 (0.16–0.77)**
≥ 50	127 (39.5)	33 (32.0)	0.61 (0.24–1.55)	24 (32.0)	0.52 (0.21–1.26)	103 (34.1)	33 (31.4)	0.61 (0.26–1.43)	15 (14.9)	**0.36 (0.15–0.84)**
Educational status										
High school or less	132 (41.1)	41 (39.8)	1	25 (33.3)	1	105 (34.8)	37 (35.2)	1	23 (22.8)	1
Matura	109 (34.0)	32 (31.1)	1.00 (0.51–1.94)	25 (33.3)	1.12 (0.56–2.21)	112 (37.1)	43 (41.0)	1.12 (0.61–2.08)	45 (44.5)	1.46 (0.75–2.82)
University degree	80 (24.9)	30 (29.1)	1.38 (0.66–2.86)	25 (33.3)	1.58 (0.78–3.22)	85 (28.1)	25 (23.8)	0.95 (0.47–1.92)	33 (32.7)	1.28 (0.63–2.59)
Marital status										
Single	117 (36.5)	44 (42.7)	1	27 (36.0)	1	82 (27.1)	32 (30.5)	1	42 (41.6)	1
Married or partnership	175 (54.5)	49 (47.6)	1.25 (0.56–2.79)	44 (58.7)	1.78 (0.80–3.94)	182 (60.3)	60 (57.1)	1.47 (0.72–2.99)	52 (51.5)	**0.49 (0.25–0.94)**
Divorced or widowed	29 (9.0)	10 (9.7)	1.18 (0.41–3.37)	4 (5.3)	1.18 (0.32–4.40)	38 (12.6)	13 (12.4)	0.59 (0.23–1.51)	7 (6.9)	0.71 (0.24–2.14)
Region of residence										
West	97 (30.2)	34 (33.0)	1	24 (32.0)	1	96 (31.8)	32 (30.5)	1	32 (31.7)	1
South	79 (24.6)	16 (15.5)	0.64 (0.29–1.44)	11 (14.7)	0.48 (0.21–1.12)	48 (15.9)	18 (17.1)	1.12 (0.52–2.41)	24 (23.8)	1.42 (0.69–2.88)
East	145 (45.2)	53 (51.5)	1.13 (0.60–2.14)	40 (53.3)	0.88 (0.46–1.66)	158 (52.3)	55 (52.4)	0.80 (0.45–1.44)	45 (44.5)	0.84 (0.47–1.52)
Residential area										
Urban	142 (44.2)	60 (58.2)	1	46 (61.3)	1	137 (45.4)	61 (58.1)	1	53 (52.5)	1
Rural ≥ 50,000	55 (17.1)	14 (13.6)	0.65 (0.28–1.51)	5 (6.7)	**0.31 (0.11–0.89)**	48 (15.9)	11 (10.5)	0.59 (0.25–1.36)	9 (8.9)	0.56 (0.23–1.34)
Rural < 50,000	124 (38.7)	29 (28.2)	0.62 (0.32–1.17)	24 (32.0)	0.54 (0.29–1.01)	117 (38.7)	33 (31.4)	0.60 (0.34–1.06)	39 (38.6)	0.93 (0.53–1.63)
Race/ethnicity										
Caucasian/white	310 (96.6)	98 (95.2)	1	71 (94.7)	1	286 (94.7)	98 (93.3)	1	91 (90.1)	1
Other	11 (3.4)	5 (4.9)	1.22 (0.33–1.17)	4 (5.3)	1.27 (0.33–4.87)	16 (5.3)	7 (6.7)	0.79 (0.28–2.21)	10 (9.9)	2.09 (0.84–5.19)
Citizenship										
Austrian	296 (92.2)	92 (89.3)	1	71 (94.7)	1	273 (90.4)	93 (88.6)	1	89 (88.1)	1
Other	25 (7.8)	11 (10.7)	0.52 (0.20–1.34)	4 (5.3)	0.38 (0.10–1.31)	29 (9.6)	12 (11.4)	1.00 (0.43–2.33)	12 (11.9)	0.99 (0.43–2.27)
Household size (including participant)										
Single	75 (23.4)	31 (30.1)	1	16 (21.3)	1	69 (22.9)	32 (30.5)	1	18 (17.8)	1
Two	102 (31.8)	41 (39.8)	1.37 (0.60–3.15)	22 (29.3)	0.77 (0.32–1.90)	115 (38.1)	35 (33.3)	0.57 (0.27–1.23)	44 (43.6)	1.84 (0.85–4.00)
Three	70 (21.8)	17 (16.5)	0.69 (0.27–1.76)	20 (26.7)	0.94 (0.38–2.31)	53 (17.5)	19 (18.1)	0.55 (0.23–1.35)	25 (24.7)	2.17 (0.89–5.25)
Four or more	74 (23.0)	14 (13.6)	**0.35 (0.12–0.98)**	17 (22.7)	0.67 (0.25–1.85)	65 (21.5)	19 (18.1)	0.39 (0.14–1.11)	14 (13.9)	0.64 (0.23–1.79)
Care taking of a child younger than 6 at home										
Yes	46 (14.3)	13 (12.6)	1	12 (16.0)	1	38 (12.6)	16 (15.2)	1	16 (15.8)	1
No^b^	173 (53.9)	49 (47.6)	1.05 (0.37–3.00)	39 (52.0)	1.17 (0.45–3.06)	159 (52.6)	63 (60.0)	0.82 (0.35–1.91)	54 (53.5)	0.79 (0.34–1.81)
No child that age	102 (31.8)	41 (39.8)	3.28 (0.97–11.1)	24 (32.0)	2.20 (0.72–6.68)	105 (34.8)	26 (24.8)	0.54 (0.29–1.48)	31 (30.7)	0.65 (0.25–1.71)
Care taking of a child between 6 and 16 at home										
Yes	60 (18.7)	22 (21.4)	1	19 (25.3)	1	52 (17.2)	25 (23.8)	1	18 (17.8)	1
No^b^	132 (41.1)	41 (39.8)	0.61 (0.22–1.69)	30 (40.0)	0.66 (0.25–1.69)	123 (40.7)	49 (46.7)	**0.42 (0.17–0.99)**	42 (41.6)	0.67 (0.28–1.58)
No child that age	129 (40.2)	40 (38.8)	**0.23 (0.07–0.72)**	26 (34.7)	0.37 (0.13–1.08)	127 (42.1)	31 (29.5)	**0.33 (0.13–0.86)**	41 (40.6)	0.69 (0.27–1.75)
Work status										
Employed full time^c^	203 (63.3)	50 (48.5)	1	52 (69.3)	1	135 (44.7)	36 (34.3)	1	58 (57.4)	1
Employed part time^c^	20 (6.2)	14 (13.6)	2.47 (0.95–6.46)	4 (5.3)	0.65 (0.19–2.24)	53 (17.6)	17 (16.2)	1.18 (0.57–2.46)	16 (15.8)	0.86 (0.42–1.74)
Retired	44 (13.7)	8 (7.8)	1.05 (0.10–11.4)	3 (4.0)	0.20 (0.01–3.29)	31 (10.3)	16 (15.2)	**9.03 (2.13–38.2)**	1 (1.0)	0.28 (0.03–3.14)
Unemployed	25 (7.8)	15 (14.6)	1.17 (0.11–12.6)	6 (8.0)	0.74 (0.05–10.7)	26 (8.6)	15 (14.3)	**7.00 (1.60–30.4)**	3 (3.0)	0.54 (0.09–3.40)
Student/in training	29 (9.0)	16 (15.5)	2.05 (0.26–16.2)	10 (13.4)	0.76 (0.07–7.74)	57 (18.9)	21 (20.0)	3.37 (0.99–11.5)	23 (22.8)	1.07 (0.31–3.77)
Short-time work										
No	181 (56.4)	32 (31.1)	1	36 (48.0)	1	156 (51.7)	46 (43.8)	1	53 (52.5)	1
Yes	46 (14.3)	34 (33.0)	**2.85 (1.40–5.76)**	21 (28.0)	**2.17 (1.04–4.51)**	40 (13.2)	14 (13.3)	0.76 (0.35–1.66)	27 (26.7)	**2.06 (1.05–4.03)**
Not employed pre-pandemic	94 (29.3)	37 (35.9)	1.88 (0.21–16.5)	18 (24.0)	1.81 (0.16–20.9)	106 (35.1)	45 (42.9)	**0.24 (0.07–0.83)**	21 (20.8)	0.49 (0.13–1.84)
Financial strain during lockdown										
No financial strain	179 (55.8)	13 (12.6)	1	31 (41.4)	1	150 (49.7)	25 (23.8)	1	48 (47.5)	1
Some	95 (29.6)	26 (25.3)	**3.35 (1.53–7.47)**	19 (25.3)	0.95 (0.46–1.92)	105 (34.8)	36 (34.3)	**2.34 (1.25–4.37)**	37 (36.6)	0.83 (0.47–1.49)
High	33 (10.3)	47 (45.6)	**16.1 (7.20–36.0)**	22 (29.3)	**2.99 (1.41–6.35)**	36 (11.9)	33 (31.4)	**6.36 (3.12–12.9)**	13 (12.9)	1.10 (0.50–2.43)
Very high	14 (4.3)	17 (16.5)	**14.6 (5.08–41.8)**	3 (4.0)	1.07 (0.27–4.45)	11 (3.6)	11 (10.5)	**7.44 (2.65–20.9)**	3 (3.0)	0.94 (0.22–3.97)
Optimism										
Low	131 (40.8)	62 (60.2)	1	29 (38.7)	1	132 (42.7)	65 (61.9)	1	45 (44.6)	1
High	190 (59.2)	41 (39.8)	0.66 (0.38–1.17)	46 (61.3)	1.32 (0.73–2.38)	170 (56.3)	40 (38.1)	**0.55 (0.33–0.94)**	56 (55.4)	1.15 (0.68–1.95)

Odds ratios in bold are statistically significant

*OR* odds ratio, *CI* confidence interval,

^a^All odds ratios are mutually adjusted for all variables in the table; the reference group are those with no change in quality of life

^b^Someone else was taking care either at home or somewhere else

^c^Includes the self-employed participants

Among women, younger age, being single and being in short-time work significantly and positively correlated with improved quality of life. Women who were married or in a partnership were less likely (OR 0.49, 95% CI 0.25–0.94) to report an increased quality of life, compared to single women. Women taking care of children between 6 and 16 years were more likely to report a decreased quality of life compared to women without children this age and compared to women not having to take care of their 6–16-year-old children. Being retired or unemployed, a high financial strain, and lower levels of optimism correlated significantly and positively with decreased quality of life in women (Table 2).

Older and more highly educated men experiencing a higher financial strain and were more likely to report increased productivity during the COVID-19 mitigation period, compared to the time before (Table 3). In contrast, short-time work seemed to be less beneficial for productivity (OR 0.52, 95% CI 0.20–1.39). Having children appeared to be associated with increased productivity in men, although men taking care of children younger than 6 years were more likely to report decreased productivity. A high financial strain also correlated with decreased productivity (Table 3).

**Table 3:** Predictors of decreased/improved productivity in the working population sub-sample (*N* = 686)

	Change in productivity									
	Men (*N* = 350)					Women (*N* = 336)				
	No change (*N* = 207)	Decreased (*N* = 96)	OR (95% CI)^a^	Improved (*N* = 47)	OR (95% CI)^a^	No change (*N* = 185)	Decreased (*N* = 111)	OR (95% CI)^a^	Improved (*N* = 40)	OR (95% CI)^a^
	*N* (%)	*N* *(%)*		*N* (%)		*N* (%)	*N* *(%)*		*N* (%)	
Age, years										
< 30	37 (17.9)	16 (16.7)	1	4 (8.5)	1	35 (18.9)	26 (23.4)	1	14 (35.0)	1
30–39	37 (17.9)	21 (21.9)	1.83 (0.72–4.66)	8 (17.0)	2.54 (0.59–10.9)	39 (21.1)	33 (29.7)	1.47 (0.65–3.34)	9 (22.5)	0.68 (0.22–2.07)
40–49	67 (32.3)	31 (32.3)	1.79 (0.72–4.42)	20 (42.6)	**4.02 (1.00**–**16.1)**	54 (29.2)	26 (23.3)	0.89 (0.39–2.04)	11 (27.5)	0.49 (0.16–1.45)
≥ 50	66 (31.9)	28 (29.2)	1.86 (0.71–4.92)	15 (31.9)	**4.56 (1.04**–**19.9)**	57 (30.8)	26 (23.4)	0.91 (0.38–2.20)	6 (15.0)	**0.20 (0.06**–**0.75)**
Educational status										
High school or less	83 (40.1)	35 (36.5)	1	8 (17.0)	1	64 (34.6)	31 (27.9)	1	18 (45.0)	1
Matura	66 (31.9)	36 (37.5)	1.30 (0.65–2.57)	12 (25.5)	2.27 (0.79–6.50)	69 (37.3)	39 (35.2)	1.09 (0.55–2.16)	12 (30.0)	**0.34 (0.13**–**0.87)**
University degree	58 (28.0)	25 (26.0)	0.83 (0.39–1.79)	27 (57.5)	**5.20 (1.92**–**14.1)**	52 (28.1)	41 (36.9)	1.85 (0.89–3.83)	10 (25.0)	0.43 (0.15–1.22)
Marital status										
Single	68 (32.9)	41 (42.7)	1	9 (19.1)	1	43 (23.2)	32 (28.8)	1	10 (25.0)	1
Married or partnership	127 (61.3)	48 (50.0)	0.47 (0.21–1.04)	36 (76.6)	1.31 (0.43–4.01)	122 (66.0)	69 (62.2)	0.88 (0.42–1.82)	27 (67.5)	1.44 (0.51–4.04)
Divorced or widowed	12 (5.8)	7 (7.3)	0.79 (0.21–2.89)	2 (4.3)	1.02 (0.15–7.00)	20 (10.8)	10 (9.0)	0.61 (0.20–1.56)	3 (7.5)	1.00 (0.18–5.45)
Region of residence										
West	66 (31.9)	34 (35.4)	1	20 (42.6)	1	61 (33.0)	39 (35.1)	1	14 (35.0)	1
South	47 (22.7)	20 (20.8)	1.10 (0.49–2.44)	4 (8.5)	**0.26 (0.08**–**0.91)**	39 (21.1)	15 (13.5)	0.64 (0.28–1.44)	8 (20.0)	0.88 (0.29–2.70)
East	94 (45.4)	42 (43.8)	1.13 (0.58–2.19)	23 (48.9)	0.69 (0.32–1.52)	85 (45.9)	57 (51.4)	0.85 (0.46–1.56)	18 (45.0)	1.00 (0.41–2.45)
Residential area										
Urban	96 (46.4)	54 (56.3)	1	21 (44.7)	1	76 (41.1)	58 (52.3)	1	19 (47.5)	1
Rural ≥ 50,000	38 (18.3)	12 (12.5)	0.50 (0.21–1.23)	4 (8.5)	0.49 (0.14–1.77)	29 (15.7)	15 (13.5)	0.85 (0.36–1.95)	5 (12.5)	0.67 (0.19–2.38)
Rural < 50,000	73 (35.3)	30 (31.2)	0.95 (0.49–1.85)	22 (46.8)	1.37 (0.62–3.03)	80 (43.2)	38 (34.2)	0.64 (0.34–1.17)	16 (40.0)	0.65 (0.28–1.51)
Race/ethnicity										
Caucasian/white	198 (95.7)	93 (96.9)	1	47 (100)	1	177 (95.7)	102 (92.9)	1	37 (92.5)	1
Other	9 (4.4)	3 (3.1)	NA	0 (0)	NA	8 (4.3)	9 (8.1)	1.55 (0.50–4.82)	3 (7.5)	1.23 (0.28–2.67)
Citizenship										
Austrian	197 (95.2)	90 (93.8)	1	43 (91.5)	1	171 (92.4)	96 (86.5)	1	38 (95.0)	1
Other	10 (4.8)	6 (6.2)	0.79 (0.22–2.82)	4 (8.5)	1.65 (0.38–7.14)	14 (7.6)	15 (13.5)	0.91 (0.36–2.31)	14 (5.0)	0.30 (0.06–1.56)
Household size (including participant)										
Single	45 (21.7)	25 (26.0)	1	5 (10.6)	1	39 (21.1)	24 (21.6)	1	10 (25.0)	1
Two	60 (29.0)	39 (40.6)	2.06 (0.84–5.05)	15 (31.9)	1.97 (0.46–8.45)	68 (36.7)	51 (46.0)	1.34 (0.61–2.96)	19 (47.5)	0.87 (0.28–2.67)
Three	59 (28.5)	14 (14.6)	0.43 (0.15–1.26)	9 (19.2)	1.16 (0.24–5.60)	39 (21.1)	15 (13.5)	0.45 (0.15–1.32)	6 (15.0)	0.45 (0.10–1.99)
Four or more	43 (20.8)	18 (18.8)	0.64 (0.20–2.05)	18 (38.3)	2.07 (0.41–10.5)	39 (21.1)	21 (18.9)	0.52 (0.16–1.73)	5 (12.5)	0.30 (0.05–1.75)
Care taking of a child younger than 6 at home										
Yes	26 (12.6)	24 (25.0)	1	11 (23.4)	1	30 (16.2)	15 (13.5)	1	5 (12.5)	1
No^b^	113 (54.6)	41 (42.7)	**0.19 (0.07**–**0.52)**	26 (55.3)	0.43 (0.14–1.33)	103 (55.7)	64 (57.7)	1.73 (0.71–4.20)	22 (55.0)	1.22 (0.32–4.63)
No child that age	68 (32.8)	31 (32.3)	**0.19 (0.06**–**0.63)**	10 (21.3)	0.37 (0.10–1.40)	52 (28.1)	32 (28.8)	1.14 (0.40–3.29)	13 (32.5)	0.86 (0.19–3.99)
Care taking of a child between 6 and16 at home										
Yes	45 (21.7)	21 (21.9)	1	16 (34.1)	1	35 (18.9)	27 (24.3)	1	7 (17.5)	1
No^b^	84 (40.6)	38 (39.6)	1.04 (0.35–3.07)	19 (40.4)	0.43 (0.14–1.33)	84 (45.4)	44 (39.7)	**0.30 (0.11**–**0.80)**	17 (42.5)	0.31 (0.07–1.40)
No child that age	78 (37.7)	37 (38.5)	0.92 (0.28–3.00)	12 (25.5)	0.37 (0.10–1.40)	66 (35.7)	40 (26.0)	0.44 (0.16–1.20)	16 (40.0)	0.54 (0.12–2.52)
Work status										
Employed full time^c^	185 (89.4)	77 (80.2)	1	43 (91.4)	1	118 (63.8)	75 (67.6)	1	36 (90.0)	1
Employed part time^c^	19 (9.2)	17 (17.7)	1.85 (0.77–4.47)	2 (4.3)	0.63 (0.12–3.43)	56 (30.2)	29 (26.1)	0.73 (0.39–1.37)	1 (2.5)	**0.04 (0.07**–**0.35)**
Retired	0(0)	0 (0)		0 (0)		2 (1.1)	1 (0.9)		0 (0)	
Unemployed	NA	NA	2.91 (0.32–26.3)^d^	NA	3.38 (0.34–33.8)^d^	NA	NA	0.83 (0.27–2.58)^d^	NA	0.94 (0.21–4.26)^d^
Student/ in training	3 (1.4)	2 (2.1)		2 (4.3)		9 (4.9)	6 (5.4)		3 (7.5)	
Short-time work										
No	159 (76.8)	52 (54.2)	1	38 (80.8)	1	152 (82.2)	72 (64.9)	1	31 (77.5)	1
Yes	48 (23.2)	44 (45.8)	1.52 (0.81–2.85)	9 (19.2)	0.52 (0.20–1.39)	33 (17.8)	39 (35.1)	**2.37 (1.25**–**4.49)**	9 (22.5)	1.02 (0.36–2.86)
Not employed pre-pandemic	NA	NA	NA	NA	NA	NA	NA	NA	NA	NA
Financial strain during lockdown										
No financial strain	120 (58.0)	16 (16.7)	1	17 (36.2)	1	101 (54.6)	32 (28.8)	1	19 (47.5)	1
Some	47 (22.7)	42 (43.7)	6.92 (3.24–14.8)	18 (38.3)	**3.45 (1.42**–**8.36)**	62 (33.5)	48 (43.3)	**2.13 (1.14**–**3.99)**	12 (30.0)	1.34 (0.51–3.52)
High	33 (15.9)	29 (30.2)	6.00 (2.68–13.4)	8 (17.0)	**3.37 (1.13**–**10.1)**	19 (10.3)	23 (20.7)	**3.29 (1.46**–**7.44)**	7 (17.5)	1.88 (0.57–6.21)
Very high	7 (3.4)	9 (9.4)	9.71 (2.72–34.5)	4 (8.5)	**7.49 (1.47**–**38.2)**	3 (1.6)	8 (7.2)	**9.60 (2.05**–**45.0)**	2 (5.0)	**10.9 (1.24**–**96.5)**
Optimism										
Low	86 (41.5)	45 (46.9)	1	14 (29.8)	1	73 (39.5)	54 (48.7)	1	14 (35.0)	1
High	121 (58.5)	51 (53.1)	1.02 (0.56–1.84)	33 (70.2)	1.34 (0.59–3.06)	112 (60.5)	57 (51.4)	0.80 (0.45–1.41)	26 (65.0)	2.00 (0.87–4.60)

Odds ratios in bold are statistically significant

*OR* odds ratio, *CI* confidence interval, *NA* not applicable

^a^All odds ratios are mutually adjusted for all predictors in the table

^b^Someone else was taking care either at home or somewhere else

^c^Includes the self-employed participants

^d^Odds ratio for participants who were retired, unemployed or students/ in training. Groups were collapsed, since numbers were very small

Older women were less likely to report increased productivity at work (OR 0.20, 95% CI 0.06–0.75) compared to younger women, as were women with a university degree compared to those less educated (high school or less) and women employed part-time compared to full-time employees. Women in short-time work were more likely to report decreased productivity compared to women not in short-time work, and a high financial strain and taking care of children between 6 and 16 years correlated significantly and positively with decrease quality of life (Table 3).

### Association of work from home with changes in quality of life and perceived productivity

Overall, participants who worked from home all the time during the COVID-19 mitigation period were more likely to report an increased quality of life (OR 3.69, 95% CI 1.86–7.29), compared to participants who were not working from home. The effect was similar for men (OR 4.11, 95% CI 1.30–13.0) and women (OR 3.33, 95% CI 1.34–8.29; Table 4). Similarly, working part of the time from home was associated with an increased quality of life (OR 2.07, 95% CI 1.09–3.91). Effect estimates had the same direction when stratifying by gender, but did not reach statistical significance. Furthermore, not working from home seemed to be associated with a decreased quality of life compared to working partially or all the time from home (Table 4). These results did not change importantly after further adjustment for changes in perceived productivity.

**Table 4:** Likelihood of positive and negative changes compared to the time pre-COVID-19 by work from home status in the working sub-sample (*N* = 686)

	Men (*N* = 350)			Women (*N* = 336)			Total (*N* = 686)		
	Working from home			Working from home			Working from home		
	No (*N* = 87)	Part time (*N* = 155)	All the time (*N* = 108)	No (*N* = 91)	Part time (*N* = 142)	All the time (*N* = 103)	No (*N* = 178)	Part time (*N* = 297)	All the time (*N* = 211)
	*N *(%)	*N *(%)	*N *(%)	*N *(%)	*N *(%)	*N *(%)	*N *(%)	*N *(%)	*N *(%)
Quality of life									
No change	60 (69.0)	109 (70.3)	58 (53.7)	59 (64.8)	83 (58.5)	54 (52.4)	119 (66.9)	192 (64.7)	112 (53.1)
Decreased	21 (24.1)	21 (13.6)	24 (22.2)	21 (23.1)	22 (15.5)	17 (16.5)	42 (23.6)	43 (14.5)	41 (19.4)
OR (95% CI)^a^	1 (Ref.)	0.51 (0.25–1.03)	1.07 (0.52–2.21)	1 (Ref.)	0.67 (0.38–1.72)	0.81 (0.38–1.72)	1 (Ref.)	0.58 (0.36–0.95)	0.94 (0.56–1.57)
OR (95% CI)^a,b^	1 (Ref.)	0.47 (0.17–1.26)	1.02 (0.36–2.91)	1 (Ref.)	0.63 (0.27–1.47)	0.72 (0.28–1.84)	1 (Ref.)	0.54 (0.30–0.97)	0.88 (0.46–1.68)
Improved	6 (6.9)	25 (16.1)	26 (24.1)	11 (12.1)	37 (26.1)	32 (31.1)	17 (9.6)	62 (20.9)	58 (27.5)
OR (95% CI)^a^	1 (Ref.)	2.39 (0.91–6.27)	4.46 (1.65–12.1)	1 (Ref.)	1.96 (0.90–4.24)	2.74 (1.23–6.12)	1 (Ref.)	2.03 (1.12–3.69)	3.18 (1.72–5.90)
OR (95% CI)^a,b^	1 (Ref.)	2.09 (0.71–6.13)	4.11 (1.30–13.0)	1 (Ref.)	1.71 (0.73–3.98)	3.33 (1.34–8.29)	1 (Rsef.)	2.07 (1.09–3.91)	3.69 (1.86–7.29)
Productivity									
No change	59 (67.8)	90 (58.1)	58 (53.7)	54 (59.3)	76 (53.5)	55 (43.4)	113 (63.5)	166 (55.9)	113 (53.6)
Decreased	21 (24.1)	47 (30.3)	28 (25.9)	25 (27.5)	47 (33.1)	39 (37.9)	46 (25.8)	94 (31.6)	67 (31.7)
OR (95% CI)^a^	1 (Ref.)	1.44 (0.78–2.68)	1.31 (0.65–2.64)	1 (Ref.)	1.22 (0.67–2.24)	1.39 (0.73–2.64)	1 (Ref.)	1.33 (0.86–2.04)	1.34 (0.84–2.15)
OR (95% CI)^a,b^	1 (Ref.)	1.83 (0.83–4.04)	1.39 (0.56–3.45)	1 (Ref.)	1.35 (0.66–2.75)	1.78 (0.82–3.84)	1 (Ref.)	1.42 (0.86–2.35)	1.48 (0.85–2.58)
Improved	7 (8.1)	18 (11.6)	22 (20.4)	12 (13.2)	19 (13.4)	9 (8.7)	19 (10.7)	37 (12.5)	31 (14.7)
OR (95% CI)^a^	1 (Ref.)	1.80 (0.70–4.63)	3.82 (1.46–9.99)	1 (Ref.)	0.94 (0.41–2.15)	0.62 (0.24–1.62)	1 (Ref.)	1.27 (0.69–2.34)	1.55(0.82–2.94)
OR (95% CI)^a,b^	1 (Ref.)	1.11 (0.36–3.38)	1.65 (0.50–5.37)	1 (Ref.)	0.72 (0.26–2.00)	0.68 (0.22–2.10)	1 (Ref.)	1.08 (0.56–2.10)	1.18 (0.58–2.45)

^a^Age-adjusted [< 30, 30–39, 40–49, ≥ 50 years]

^b^Additionally adjusted for gender [binary] (in not-gender stratified models), race (white/Caucasian, other), citizenship (Austrian, other), educational attainment [High school or less, Matura(University entry exam), university degree], region of residence (west, south, east), area of residence (urban area, rural with < 50,000 inhabitants, rural with ≥ 50,000 inhabitants), marital status (single, married or partnership, divorced or widowed), number of children [none, one, two, three or more], taking care of children younger than 6 years at home (yes, no, no child that age), taking care of children between 6 and 12 years at home (yes, no, no child that age), household size (one, two, three, four, five or more persons), short-time work (binary), job loss [binary], work status [full time employed (including self-employed), part time employed (including self-employed)], financial strain during lockdown (None, some, high, very high) and dispositional optimism (low, high)

In contrast, participants working part or all of the time from home appeared more likely to report decreased productivity (vs. not working from home, partially: OR 1.42, 95% CI 0.86–2.35; all the time: OR 1.48, 95% CI 0.85–2.58) (Table 4).

## Discussion

Overall, quality of life and perceived productivity improved in a sizeable segment of the Austrian population sample during the COVID-19 mitigation period. Simultaneously, these factors decreased in an even larger part of the sample. Working from home during the mitigation period was associated with an improved quality of life, consistent with prior reports that Austrians expressed a preference to work from home (TQS Research & Consulting 2020), but this did not correspond to increased perceived productivity. Furthermore, our results suggested that changes in perceived productivity did not drive the association of working from home with increased quality of life.

Our results and interpretations are limited by not having assessed potentially informative aspects of employment history or the working environment and related conditions at home. For example, we did not collect information on income or whether individuals worked from home prior to the 50-day mitigation period, some, although probably very few, might already have worked from home prior to the pandemic. We also did not assess if working from home was also coupled with greater working hour flexibility. According to Hill et al. ‘Work-at-home should be coupled with perceived schedule flexibility to maximize benefits’ (Hill et al. 2010). Measures to assess changes in quality of life and perceived productivity were not validated. Furthermore, we could not quantify the changes in self-reported quality of life and productivity, and changes in one’s perceived productivity may not correspond to changes in employer-assessed productivity. Participants might have interpreted the term “productivity” in various ways and results must, therefore, be interpreted cautiously. That circumstances surrounding the pandemic might have altered the perception, definition, and determinants of productivity limits the generalizability of our findings. Furthermore, change in quality of life, a multidimensional concept, was assessed with a single question adding to the limited generalizability of our findings, as dimensions within the concept of quality of life might have changed differentially during the pandemic. The cross-sectional design of our study precludes causal inferences, and non-differential misclassification could have led to underestimates in our results. Furthermore, while reverse causation appears unlikely, the reported association between working from home and quality of life and perceived productivity could be confounded by such factors as type of occupation, commuting distance, general job satisfaction, (Bhattarai 2020) and pre-pandemic work arrangements, for which we could not properly adjust.

Overall, a decrease in quality of life and perceived productivity was reported more frequently than an increase in our sample suggesting that the first mitigation period affected the life of the Austrian population negatively, although some individuals appeared to benefit from the introduced measures. Pieh et al. (2020) reported that the mental health burden during the mitigation period was alarmingly high among young Austrian adults and suggested that this could potentially be explained by their higher occupational uncertainty and larger restrictions in their daily lives. In the present study, most younger participants reported no change in quality of life, with similar proportions reporting a decrease or an increase in quality of life. However, middle-aged and older adults were notably more likely than younger participants to report no change; hence, the mitigation measures appear to have affected the quality of life of younger Austrians more than other age groups, both positively and negatively. That these effects on quality of life might differ depending on age was recently corroborated by a Belgian survey among young physicians, where 56% reported a positive impact of the COVID-19 crisis on their life (Degraeve et al. 2020).

Pieh et al. (2020) also reported that the mental health of women in Austria was more negatively affected during the mitigation period. In our study, the reported decrease in quality of life was almost identical across gender, and women more frequently reported an improved than a decreased quality of life. Interestingly, being married or in a partnership appeared to correlate with a positive change in quality of life in men, whereas for women, the opposite effect was observed; women who were married or in a partnership were less likely than single women to report an increased quality of life. One explanation for this observation could be that women might have had to shoulder a bigger part of the mitigation period´s consequences when in a partnership, especially in families with children. And indeed, although with limited power, our results are suggestive for slightly stronger effects among women in partnerships with children, compared to single women or women in partnerships without children. A German COVID-19 survey showed that even today, women are still carrying most of the burden of childcare, household chores, and care for the elderly (Czymara et al. 2020; Hans-Böckler-Stiftung 2020). In Spain, the closure of schools and daycares increased the time women spent on home schooling and domestic care, whereas males increased their contribution to housework only slightly (Farré et al. 2020).

In the present study, higher optimism correlated with lower likelihood of decreased quality of life in women. However, optimism did not seem to be linked with a higher likelihood of reporting an improved quality of life. One potential explanation for this observation might be related to resilience (Aburn et al. 2016), which is an important trait during challenging situations, with optimism as an essential contributor (Lee et al. 2013). However, more research is needed to elucidate these potential associations.

Surprisingly, high financial strain correlated with improved quality of life and perceived increased productivity in men. Less work could lead to an improved quality of life (e.g., more time for hobbies) but also to a higher financial strain, which in turn could motivate people to work more or could influence their perceived productivity. In a similar manner, our observations of more frequently reported increases in quality of life for participants in short-time work arrangements or those working from home may be explained by more flexibility in the attribution of available time throughout the day and by simply having more time available to attribute to certain activities (Hill et al. 2010). Working partially or all the time from home implies that commuting is no longer part of daily life. In Austria, this implies that about 53% of the working population could free up 30 min to 2 h every day (STATISTIK AUSTRIA 2017) and allot the available time to other more pleasurable or productive activities. For example, an ability to allot time to an activity at a preferable time (having lunch with the family, meeting friends, doing sports, etc.) may be a key element of quality of life that enhances healthy lifestyle behaviors and thereby contributes to a stronger sense of well-being in the face of co-existing challenges. However, these findings must be interpreted with caution as they may be limited to societies and situations where working from home is a choice, rather than mandated by a pandemic.

This hypothesis is corroborated by other evidence. For example, a study into the COVID-19 mitigation period in Chile revealed that 60% of the population were preparing food at home more frequently than before, 33.7% ate more healthily and 23% were physically more active (Reyes-Olavarría et al. 2020). In a French sample, 18.7% increased their level of physical activity during the mitigation period and 23% reported weight loss. More favorable lifestyle behaviors correlated more strongly with education level, income, and working from home. The authors also noted that a mitigation period enabled an important segment of the population to improve their nutritional behavior in potentially sustainable ways (e.g., post mitigation period) (Deschasaux-Tanguy et al. 2020). In a qualitative study, participants reported negative long-term behavioral changes after a quarantine (not COVID-19 related). The authors suggested that a similar pattern of longer term sustained change might apply to positive changes as well (Brooks et al. 2020). Lack of time is one of the most important barriers to adopting and maintaining a healthy lifestyle, especially in working populations (Kearney and McElhone 1999; Spiteri et al. 2019). Therefore, gaining time through more flexible working hours and workplace arrangements could have important positive implications for society and may explain the present findings.

Overall, a deeper understanding of side effects of more flexible working conditions is needed. In our study, working from home seemed more often to be associated with decreased rather than increased perceived productivity. A qualitative study into the COVID-19 mitigation period in April 2020 in Indonesia supports that working from home improved work-life balance, workplace and time flexibility and diminished participants’ discomfort from otherwise working under constant supervision. However, participants also reported decreased motivation, increased distraction, and difficulties communicating with colleagues and managers (Mustajab et al. 2020). In a survey of 51 Italian administrative officers who had started to work remotely during the beginning of the COVID-19 crisis, 39.2% of participants indicated lower and 29.4% higher productivity compared to before they started to work from home. 62.7% wanted to continue working from home occasionally or all the time, while the lack of interaction with colleagues was the main reason for 31.4% of participants to indicate the opposite. The rest (5.9%) indicated distractions as the main reason for not wanting to continue to work from home (Moretti et al. 2020). The ability to concentrate was shown to be an essential influence on perceived productivity (Maarleveld and de Been 2014). Some factors, e.g., having to take care of children at home or having to sit on a non-ergonomic chair all day long, could be unique to the COVID-19 mitigation period. Moreover, worldwide productivity—in almost every way—reached a low during the COVID-19 mitigation period, likely independent of the concrete place of work (World Bank 2020).

In their 2017 report on telework, Eurofound and the International Labour Office concluded that telework has mainly positive effects on individual performance, explained in part by longer working hours and a higher ability to concentrate due to fewer interruptions (Eurofound 2017). Yet, working from home had not been widely adopted in Europe before the emergence of COVID-19 (Eurofound 2017). In a survey administered in 75 countries, working from home and perceived schedule flexibility were related to less work-life conflict. However, women with children aged five or younger were more likely to report work difficulties when working from home compared to women who did not primarily work from home (Hill et al. 2010). This result points towards a key nuance when discussing benefits and downsides of working from home: effects differ substantially across different subgroups. In our study, for example, older and more highly educated men were more likely to report an increased perceived productivity, whereas this was not seen in women of similar age and education level. Recently, women in science reported a substantial decline in time devoted to research (Myers et al. 2020), and women were less likely to report job satisfaction compared to men when working from home during the pandemic (Bhattarai 2020). In our study, inequalities also surfaced when looking at the distribution of working from home by gender, age, and educational status. Working from home was much more frequent among higher educated participants, suggesting that less educated participants were less likely to benefit from any improvements associated with working from home. In our study, not working from home during the mitigation period seemed to be associated with a decreased quality of life. Other studies reported that changes in lifestyle differed by income (Deschasaux-Tanguy et al. 2020) and that economically vulnerable groups experienced more negative consequences from the crisis (Hans-Böckler-Stiftung 2020). These inequalities and the ones referred to previously might partially be explained by the unequal distribution of organizational and occupational factors such as job insecurity, uncertainty of the future, and long periods of isolation (Giorgi et al. 2020) that can influence the mental response of workers during the pandemic. While in theory the implementation of resilience training interventions targeted at vulnerable parts of the population could bring some relief (Giorgi et al. 2020; Wang et al. 2020), especially when interventions are adaptable for local needs and are introduced to an environment with effective communication and safe and supportive learning environments (Pollock et al. 2020), their practicability and usefulness remains open to debate. To our knowledge, ours is the first study to describe associations between working from home during a COVID-19 mitigation period and quality of life and perceived productivity at a population level. The results from our survey of the Austrian population might not be generalizable to other populations, and the generalizability to the whole Austrian population might also be limited by drawing participants from an Online-Panel. Furthermore, results might not be generalizable to pre- and post-pandemic conditions considering the exceptional characteristics of the first mitigation period in Austria compared to “normal” times. In addition, we captured merely the 50 days of the COVID-19 mitigation period in Austria, warranting further examination of the long-term associations of working from home with quality of life. Lastly, studies are needed to evaluate the implications of our results in the context of the One Health paradigm, i.e., how exactly does more workplace flexibility reduce our ecological footprint?

The ongoing pandemic is an opportunity for researchers and companies alike to further investigate effects of working from home on the employee´s life. Future longitudinal studies will ideally investigate both short-term and long-term associations of working from home with key indicators of the employee’s quality of life, perceived productivity, and objectively measured productivity to clarify the positive and negative repercussions of working from home for employees and employers, and ultimately to inform policy makers. Results for different subgroups and stakeholders are needed (since outcomes might be positive for one group but negative for another), and barriers and facilitators for a positive impact of working from home on quality of life should be identified, including the influence of sociodemographic factors and the working environment/conditions at home. Studies should also avail of the opportunities to incorporate further transitions as the pandemic resolves. Such further research holds the potential to inform beneficial public policy to minimize longer term negative consequences of COVID-19 prevention measures and better understand and mitigate existing societal inequalities and their implications for family and professional life, for stakeholders, for the environment, and ultimately for public and One Health. Society should try to use the momentum, in this case the recently experienced transition to more flexibility of workplace and working hours for employees, to improve life and environmental conditions in the future.

## Supplementary Information

Below is the link to the electronic supplementary material.

Supplementary file1 Changes in productivity at work during the COVID-19 mitigation period in the entire working population sub-sample and by work from home status (PDF 127 KB)

## Data Availability

Data are available by contacting the corresponding author and following acceptance by the contributing centers.

## Code availability

Codes are available by contacting the corresponding author and following acceptance by the contributing centers.
