# Supplementary figures and images for: Working from home, quality of life, and perceived productivity during the first 50-day COVID-19 mitigation measures in Austria: a cross-sectional study
Source: Int Arch Occup Environ Health. 2021 Apr 20;94(8):1823–37. doi: 10.1007/s00420-021-01692-0 (PMC8056371; doi:10.1007/s00420-021-01692-0)

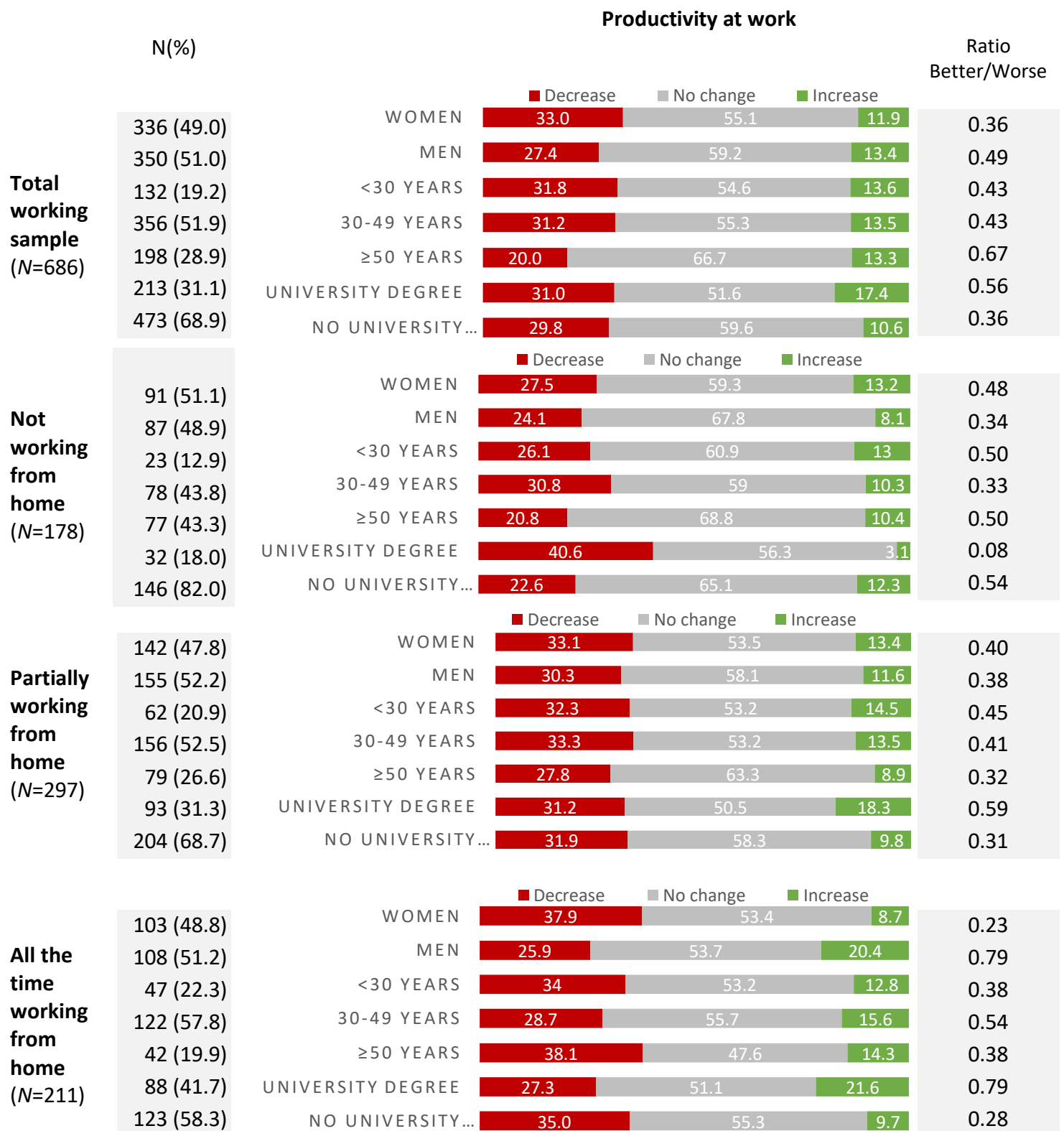

Supplement: Supplementary file 1 — Supplementary file1 Changes in productivity at work during the COVID-19 mitigation period in the entire working population sub-sample and by work from home status (PDF 127 KB) [file 420_2021_1692_MOESM1_ESM.pdf]
